# Development of Multispecies Recombinant Nucleoprotein-Based Indirect ELISA for High-Throughput Screening of Crimean-Congo Hemorrhagic Fever Virus-Specific Antibodies

**DOI:** 10.3389/fmicb.2019.01822

**Published:** 2019-08-23

**Authors:** Neha Shrivastava, Ambuj Shrivastava, Sandeep M. Ninawe, Shashi Sharma, Jyoti S. Kumar, Syed Imteyaz Alam, Amit Kanani, Sushil Kumar Sharma, Paban Kumar Dash

**Affiliations:** ^1^Division of Virology, Defence Research and Development Establishment, Gwalior, India; ^2^Division of Biotechnology, Defence Research and Development Establishment, Gwalior, India; ^3^Office of Deputy Director of Animal Husbandry, FMD Typing Scheme, Ahmedabad, India

**Keywords:** Crimean-Congo hemorrhagic fever, antigen, protein purification, IgG, seroepidemiology, diagnosis

## Abstract

Crimean-Congo hemorrhagic fever (CCHF) is a re-emerging zoonotic viral disease prevalent in many parts of Asia, Europe, and Africa. The causative agent, Crimean-Congo hemorrhagic fever orthonairovirus (CCHFV), is transmitted through hard ticks. Tick vectors especially belonging to the *Hyalomma* species serve as the reservoir and amplifying host. The vertebrate animals including sheep, goat, and bovine act as a short-lasting bridge linking the virus and ticks. CCHFV causes fatal hemorrhagic fever in humans. Humans are usually infected with CCHFV either through the bite of infected ticks or by close contact with infected animals. Immunological assays, primarily enzyme-linked immunosorbent assay (ELISA) using whole viral antigen, are widely used for serosurveillance in animals. However, the whole virus antigen poses a high biohazard risk and can only be produced in biosafety level 4 laboratories. The present study focuses on the development and evaluation of safe, sensitive, and specific IgG indirect enzyme-linked immunosorbent assay (iELISA) using recombinant nucleoprotein (NP) of CCHF virus as an antigen. The codon-optimized NP gene sequence was synthesized, cloned, and expressed in pET28a+ vector. The recombinant NP was purified to homogeneity by affinity chromatography and characterized through Western blot and MALDI-TOF/MS analysis. The characterized protein was used to develop an indirect IgG microplate ELISA using a panel of animal sera. The in-house ELISA was comparatively evaluated vis-à-vis a commercially available ELISA kit (Vector-Best, Russia) with 76 suspected samples that revealed a concordance of 90% with a sensitivity and specificity of 79.4 and 100%, respectively. The precision analysis revealed that the assay is robust and reproducible in different sets of conditions. Further, the assay was used for serosurveillance in ruminants from different regions of India that revealed 18% seropositivity in ruminants, indicating continued circulation of virus in the region. The findings suggest that the developed IgG iELISA employing recombinant NP is a safe and valuable tool for scalable high-throughput screening of CCHFV-specific antibodies in multiple species.

## Introduction

Crimean Congo hemorrhagic fever orthonairovirus (CCHFV), an emerging tick-borne virus, is considered as a biothreat agent because of its potential to cause deadly hemorrhagic fever. A tick-borne virus, CCHFV belongs to the family *Nairoviridae* and genus *Orthonairovirus* (Adams et al., 2017). It is endemic in different parts of the world including Asia, Africa, and Europe with a fatality rate up to 80% (Ergönül, 2006). In humans, it causes acute hemorrhagic illness, leading to hypovolemic shock and death in extreme cases. CCHFV is primarily transmitted to humans through the bite of an infected tick. In animals, CCHF is asymptomatic and does not cause any disease but is a major threat to humans especially to those who are in close contact with infected animals particularly in farms, clinic, and abattoir (Mostafavi et al., 2017).

The genome of CCHFV is triple segmented, consisting of small (S), medium (M), and large (L) segments. Electron microscopy depicts viral particles of 90–100 nm in diameter (Zivcec et al., 2016). The open reading frame (ORF) of the S segment encodes a 482-amino-acid-long nucleocapsid protein that is highly conserved in nature and is an attractive target for development of diagnostics and vaccine (Burt et al., 1994; Carter et al., 2012; Vanhomwegen et al., 2012).

Many of the CCHF cases were reported from remote settings, where there is a lack of proper detection facility that leads to underreporting. Limited serosurveillance studies have shown high IgG positivity in livestock in different parts of endemic areas including India (Mourya et al., 2015). Ruminants play a crucial role in providing blood meal to ticks, their transportation across different territorial regions, and transmitting virus to naïve ticks and humans (Mertens et al., 2015; Spengler et al., 2016). Efforts are needed to detect the infection burden in animals using a safe, stable, and sensitive screening test that can be easily accessible in rural health care facilities.

The laboratory detection of CCHFV relies on virus isolation, RT-PCR, and serology. Virus isolation and RT-PCR are limited to biosafety level 4 (BSL-4) laboratory and highly sophisticated laboratory, respectively, having technical expertise (Al-Abri et al., 2017). Further, serological assays, viz., hemagglutination inhibition and complement fixation tests, suffer from various issues including reproducibility and sensitivity (Casals and Tignor, 1974). The currently used serological tests include immunofluorescence (IFA) and enzyme-linked immunosorbent assay (ELISA), though IFA demands specialized equipment, technique, and complexity in data interpretation, whereas ELISA can be performed in laboratory with minimum resources (Vanhomwegen et al., 2012). The widely used serological assays rely on whole virus as the antigen, primarily inactivated viral lysate, or hyper immune mouse ascitic fluid, which is used to capture antibodies (Mourya et al., 2012). Such antigen poses a high biohazard risk and their production is limited to maximum containment facilities (BSL-4), which are limited in the developing world.

The recombinant protein-based antigen is slowly replacing the viral diagnosis over the last few decades (Cuzzubbo et al., 2001; Álvarez-Rodríguez et al., 2012; Souza et al., 2012; Saxena et al., 2013). Bacterial expression systems are the preferred method of choice due to their inexpensive scale up and ease of utility (Babaeipour et al., 2010; Huang et al., 2012). The ease and reproducibility of antigen production make these antigens reliable for commercialization. Attempts were made to develop IgG ELISA for CCHF using recombinant nucleoprotein (NP) in baculovirus (Saijo et al., 2002; Samudzi et al., 2012). However, such expression systems are expensive, need to reinfect with fresh culture for each round of protein synthesis, and thus are inefficient for commercial-scale production. Recombinant NP protein was also attempted to express in prokaryotic system but protein instability was the major concern of the reported system (Liu et al., 2014). Recently, HRP-conjugated recombinant NP-based ELISA was developed for IgG detection, but the HRP-conjugated antigens are liable to lose their epitopes during conjugation and thus lack sensitivity; also, the stability of HRP-conjugated antigen is questionable until experimentally proven (Ge et al., 2017; Emmerich et al., 2018; Sas et al., 2018). Earlier, Schuster et al. (2016) reported the development of a competitive ELISA format using monoclonal antibodies against NP for serosurveillance. However, the involvement of specific monoclonal antibody and the competitive format make the process complex and costlier. The production of recombinant protein in the eukaryotic system is complex and expensive compared to the bacterial system. Keeping in view the above facts, the present study was envisaged to produce a safe and stable recombinant antigen in the prokaryotic system for the development of IgG iELISA assay. Recombinant NP was produced under native conditions in order to express its conformational epitopes to recognize all the possible antibodies. The soluble r-NP was used as a diagnostic intermediate for development of IgG iELISA. The assay was further evaluated with commercial system, and concordance, sensitivity, along with specificity were determined. Both intra- and inter-assay variations were also analyzed to study the precision of assay. Finally, the assay was applied for screening of animal sera from different regions in India for epidemiological serosurveillance.

## Materials and Methods

### Media, Chemicals, and Solutions

*Escherichia coli* strain DH5α was procured from Invitrogen, United States. *E. coli* host strain BL21(DE3) for expression of plasmid was purchased from Thermo Fisher Scientific, United States. The cloning vector pUC57 (amp^r^) and expression vector pET28a+ (kan^r^) were purchased from Novagen, United States. Ni-NTA superflow resin was procured from Qiagen, Germany. Protease inhibiting cocktail (Sigma-Aldrich) and TB were from HiMedia. Secondary HRP conjugates (anti-sheep HRP, anti-goat HRP and anti-bovine HRP) and HRPO substrate 3,3′,5,5′-tetramethylbenzidine (TMB) were procured from Sigma-Aldrich, United States. VectoCrimea CHF IgG ELISA kit was purchased from Vector-Best, Russia.

### Construction of Recombinant Plasmid

The ORF encoding NP of CCHF virus from Indian isolate ID NIV 112143 (GenBank Accession no. JN572089) was codon optimized for *E. coli* and custom synthesized from M/s Bio Basic Inc., Canada. The custom synthesized gene (1458 bp) was confirmed by nucleotide sequencing and cloned in pUC 57 cloning vector.

### Sub-Cloning of Recombinant NP Gene in pET28a+ Vector

Both recombinant plasmid of NP in pUC57 cloning vector and pET28a+ expression vector were digested with *Bam*HI and *Hin*dIII restriction enzymes (RE) at 37°C for 1 h. The insert (NP) and the vector (pET28a+ expression vector) were purified using agarose gel and ligated. The ligated product was transformed into competent *E. coli* DH5α cells as per standard protocol. Transformed positive clones were selected by RE digestion and colony lysis PCR. The recombinant plasmid was isolated and further transformed into *E. coli* BL21 (DE3) cells for expression. The recombinant transformed clone was also confirmed by RE digestion, colony lysis PCR, and nucleotide sequencing.

### Expression and Localization of Recombinant Protein

The logarithmic phase culture of positive colony was induced with varying concentrations of isopropyl-β-D-thiogalactopyranoside (IPTG) (0.5, 1, 1.5, and 2 mM) in Terrific broth (TB) for different time periods (1–4 h). Further, another lot was induced at 18°C for 18 h. The localization of recombinant protein was identified through sonication of cell suspension followed by centrifugation at 18,600 × *g* for 30 min at 4°C. The expression profile was studied by 10% SDS-PAGE.

### Purification of Recombinant Nucleoprotein Under Native Conditions

Following induction, cells were harvested by centrifugation at 4,000 × *g* for 20 min. Harvested cells were then resuspended in lysis buffer (50 mM NaH_2_PO_4_, 300 mM NaCl, and 10 mM imidazole, pH 8.0) supplemented with phenylmethylsulfonylfluoride (PMSF), lysozyme, and protease inhibiting cocktail (Sigma-Aldrich, United States) and incubated at 4°C for 30 min, followed by sonication at 9 pulse on/off using microprobe at 40% frequency of sonicator (Sonics, United States) for 30 min. The sonicated culture was centrifuged at 10,000 × *g* for 20 min. The supernatant was filtered with a 0.45-μm filter and loaded on the pre-equilibrated polypropylene column containing Ni-NTA slurry for 2 h at 4°C for efficient binding. After passing the supernatant through a column, it was washed with 10 bed volumes of wash buffer (50 mM NaH_2_PO_4_, 300 mM NaCl, and 20 mM imidazole, pH 8.0) to remove unbound protein. Later on, the bound protein was eluted through elution buffer (50 mM NaH_2_PO_4_, 300 mM NaCl, and 250 mM imidazole, pH 8.0). Eluted protein was concentrated using an Amicon Ultra centrifugal filter device with a 30-kDa MWCO membrane. Purified protein was dialyzed overnight against dialysis buffer (50 mM NaH_2_PO_4_ and 300 mM NaCl). Protein was analyzed on 10% SDS-PAGE followed by staining with Coomassie Brilliant Blue. The concentration of purified protein was estimated by bicinchoninic acid assay (Thermo Fisher Scientific, United States) and stored at −80°C with 5% glycerol to enhance the stability of recombinant NP.

### Characterization of r-NP Through Western Blot Analysis

Characterization of r-CCHF-NP was carried out using commercial rabbit polyclonal antibody against NP of CCHFV (Abcam, United States) and anti-His antibody (Sigma-Aldrich, United States) with Western blot. The purified r-NP protein was resolved on 10% SDS and transferred to nitrocellulose membrane. Membranes were blocked with 2% SMP for 2 h, followed by washing with 1 × PBS. Then, the membranes were incubated with 1:500 dilution of commercial polyclonal antibody and 1:1,000 dilution of anti-His antibody diluted in 1% SMP overnight, respectively. After washing thrice with 0.1% PBST, membranes were incubated with their respective HRP conjugate (anti-rabbit and anti-mice HRP conjugate). The conjugates were diluted in 1% Skim milk powder (1:1000) for 2 h followed by washing with 0.1% PBST. The membrane was finally developed using 3,3-diaminobenzidine (DAB) as substrate and H_2_O_2_.

### In-Gel Protein Digestion

Gel pieces were excised from the SDS-PAGE and destained by three washes of 50% ACN/50 mM NH_4_HCO_3_ for 30 min each at room temperature. The protein was subjected to reduction with 10 mM DTT followed by an alkylation with 50 mM iodoacetamide. Gel pieces were dried after treatment with ACN and 100 ng of trypsin in 50 mM NH_4_HCO_3_ was added to the gel pieces. Tryptic digestion was carried out overnight at 37°C and the peptides were extracted with 60% ACN and 0.1% TFA. The peptides were vacuum dried and resuspended in 0.5% TFA before the MALDI-MS/MS analysis.

### MALDI-TOF-TOF Analysis of Recombinant Nucleoprotein of CCHF

The recombinant NP of CCHF was identified by Applied Biosystems 4800 plus MALDI TOF/TOF Analyzer (AB Sciex, United States) using the tryptic digest of the protein. The digested peptides were spotted onto the target plate following mixing with 1:1 volume of CHCA matrix solution (10 mg/ml). MS mass spectrum was recorded in the reflector positive mode using a laser with accelerated voltage of 2 kV (200 Hz repetition rate with a wavelength of 355 nm). A default calibration was applied for 13 calibration points on 384-well MALDI plate, using a six-component peptide standard in a mass range of 905–3,660 Da. The MS/MS mass spectra were acquired by the data-dependent acquisition method by collision-induced dissociation (CID) at 1 kV voltage. MS/MS fragment ions were generated for 20 strongest precursors selected between the *m*/*Z* range of 850–4,000 Da and filtered with a signal-to-noise (S/N) ratio >20 from one MS scan. The precursor ions were selected for fragmentation by a timed ion selector (TIS), using air as collision gas at 1 kV energy and a recharge pressure threshold of 1.5e-006. MS and MS/MS spectra were accumulated for at least 1,200 and 1,600 laser shots, respectively. Peak list was generated using the 4000 Series Explorer Software v. 3.5 (Applied Biosystems) wherein the MS/MS peaks were selected on the basis of a S/N ratio greater than 10. The data thus obtained were explored employing Protein Pilot version 4.0 (Applied Biosystems) and MASCOT 2.0 search engine (Matrix Science, London, United Kingdom). Finally, the peak list was searched against all entries at the non-redundant protein sequence database of NCBI with 16,338,050 sequences using the following search parameters: mass tolerance of 50 ppm for precursor ion and ±0.6 Da for fragment ion with +1 charge state, trypsin digestion with one missed cleavage, and variable modifications of oxidation of methionine and carbamidomethylation of cysteine.

### Recombinant Nucleoprotein-Based IgG ELISA

Recombinant CCHF NP protein was tested for its antigenicity in terms of recognition by anti-CCHFV antibody in bovine, sheep, and goat serum samples. For standardization of indirect IgG ELISA, each well of a 96-well maxisorp microtiter plate (Nunc, Denmark) was coated with 300 ng of recombinant CCHF r-NP protein in carbonate–bicarbonate buffer (pH-9.6). Following overnight incubation, the plate was washed thrice with PBST (PBS containing 0.05% Tween 20) and blocked with 5% SMP overnight at 4°C. Test sera were heat inactivated at 56°C for 30 min prior to dilution (1:1,000) in 1% SMP in 0.1% PBS-T. The diluted sera were added to each well and incubated at 37°C for 60 min. The plate was washed with wash buffer (0.1% PBST) five times and then incubated with 1:10,000 dilution of respective HRP-conjugated antibody (Sigma-Aldrich, United States) in 0.05% PBST at 37°C for 60 min. This was followed by washing five times with wash buffer and the reaction was developed by adding 100 μl of TMB (Sigma-Aldrich, United States). Following incubation at room temperature for 10 min, the reaction was stopped using 100 μl of 1N HCl as stop solution and the absorbance was recorded at 450 nm using an ELISA plate reader (BioTek, United States). Thirty negative control sera from healthy animals were included for the standardization of IgG iELISA. All these parameters including coating, dilution of serum and conjugate, washing, and incubation were optimized by taking into account the S/N ratio.

### Comparative Evaluation of In-House ELISA With Commercial ELISA Kit

A panel of 76 animal samples from the CCHF-affected region of Gujarat, India, were tested in parallel with in-house iELISA and commercial VectoCrimea CHF IgG ELISA kit (Vector-Best, Russia). The protocol standardized above was used for in-house iELISA, whereas that recommended by M/s Vector-Best was followed during performance of VectoCrimea IgG ELISA kit, with optimization of animal conjugates through checkerboard titration.

### Data Analysis

Data were analyzed with SigmaStat 4.0 software by applying confidence of interval at 95%. Vector-Best ELISA was considered as reference test in this study. The cutoff value of in-house iELISA was determined employing receiver operating characteristic (ROC) curve and area under the curve (AUC) analysis. The ROC curve of in-house and VectoCrimea was eventually compared (Hewson et al., 2004) to determine the sensitivity, specificity, positive predictive value (PPV), and negative predictive value (NPV) of the in-house iELISA. The correlation and percent agreement between the two assays were calculated using Spearman correlation coefficient. The inter-rater agreement of two assays was measured using Cohen’s kappa coefficient.

### Assay Reproducibility

Robustness of the assay is a crucial parameter for validation of assay, which is defined as the ability of a method to remain unaffected by small variation (Chan et al., 2013). Intra-assay variation (i.e., variation of replicates in one experiment), inter-assay variation (i.e., variation between experiments performed on different days by same operator), and inter-operator variation (i.e., variation between experiments performed by different operators) were performed to study the validation and reproducibility of the assay in different sets of conditions. Intra-assay was performed to monitor the variation in replicates within an assay. Each of six samples (three positive and three negative) were tested in five replicates within an assay, and intra-assay coefficient of variation (standard deviation/average × 100) was calculated. Kruskal–Wallis and repeated measures (RM) one-way ANOVA were calculated for intra-assay. The test was then performed using these calibrators in duplicate on eight different days for inter-assay variation. A total of 16 replicates were tested using the in-house IgG iELISA. The inter-operator assay variation was performed at different laboratories with different operators using the panel of calibrators of low, medium, and high IgG positive samples along with IgG negative sera samples. The values thus obtained were statistically analyzed with Kruskal–Wallis, one-way ANOVA, and percent coefficient of variation for comparing two or more groups.

### Serosurveillance for CCHF in Animal Species

In-house IgG iELISA was then applied to screen a total of 640 animal samples including sheep (*n* = 198), goat (*n* = 249), and bovine (*n* = 193), collected from different Indian states such as Gujarat, Jammu and Kashmir, and Rajasthan.

## Results

### Cloning, Expression, and Solubility Optimization of CCHF NP Gene

**FIGURE 1:**
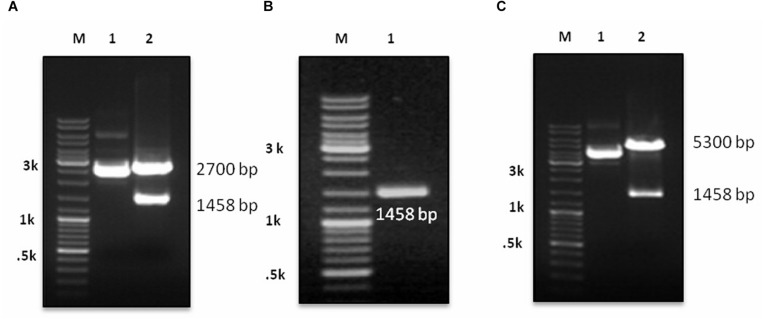
**(A)** M: DNA Marker (GeneRuler DNA Ladder, Cat. # SM0333, Thermo Fisher Scientific), lane 1: undigested pUC 57 with cloned gene, lane 2: digestion of recombinant plasmid with *Bam*HI and *Hin*dIII revealing release of insert. **(B)** M: DNA Marker; lane 1: LMP purified gene insert (1458 bp). **(C)** M: DNA Marker; lane 1: ligated gene insert (1458 bp) with pET28a+ expression vector, lane 2: confirmation of ligation through RE digestion of recombinant plasmid with *Bam*HI and *Hin*dIII, revealing fragment 5300 bp of vector and 1458 bp of gene insert.

**FIGURE 2:**
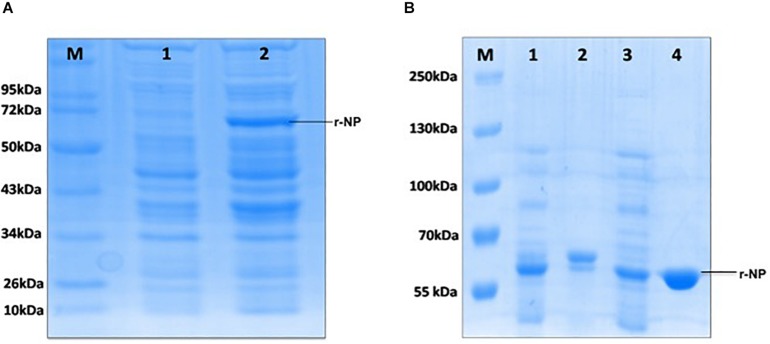
**(A)** M: Protein molecular weight marker (Cat. # SM0661, Fermentas); lane 1: uninduced BL21 (pET28a+), lane 2: induced BL21 (pET28a+) with 1 mM IPTG. **(B)** M: Protein molecular weight marker (Cat. #26619, Thermo Fisher Scientific); lane 1: induced bacterial lysate in insoluble form, lane 2: purified protein from insoluble lysate (under denaturing condition), lane 3: induced bacterial lysate in soluble form, lane 4: purified protein from soluble lysate (under native condition).

The synthetic gene (1,458 bp) coding for CCHF NP cloned in pUC57 vector was used to release the insert using *Bam*HI and *Hin*dIII RE (Figure 1A). The insert was gel purified revealing a size of 1458 bp (Figure 1B). The insert was cloned into pET28a+ expression vector at *Bam*HI and *Hin*dIII sites and transformed in *E. coli* BL21 (DE3) cells. The presence of insert was confirmed through RE analysis, which revealed the release of a 1,458-bp insert (Figure 1C). The expression of NP from positive clone was analyzed in SDS-PAGE. A clear band of 56 kDa was observed in induced culture compared to uninduced cells (Figure 2A). Optimization of induction profile with varying IPTG concentration led to an optimum IPTG concentration of 1 mM (Supplementary Figure S1A). SDS-PAGE analysis revealed that the fraction of r-NP was found in insoluble form as inclusion bodies in post-induced culture up to 4 h at 37°C. To shift the expression to soluble form, conditions were further optimized by lowering the temperature of post-induction (from 36 to 18°C) (Figure 2B and Supplementary Figure S1B). After optimization, transformed BL21 (DE3) cells were grown in TB broth supplemented with 1% glycerol (Vagenende et al., 2009) and 50 μg/ml kanamycin. The expression of mid-log phase culture was induced by IPTG and grown post-induction at 18°C overnight that revealed expression of r-NP of 56 kDa size in soluble form.

### Purification of Recombinant CCHF Nucleoprotein

**FIGURE 3:**
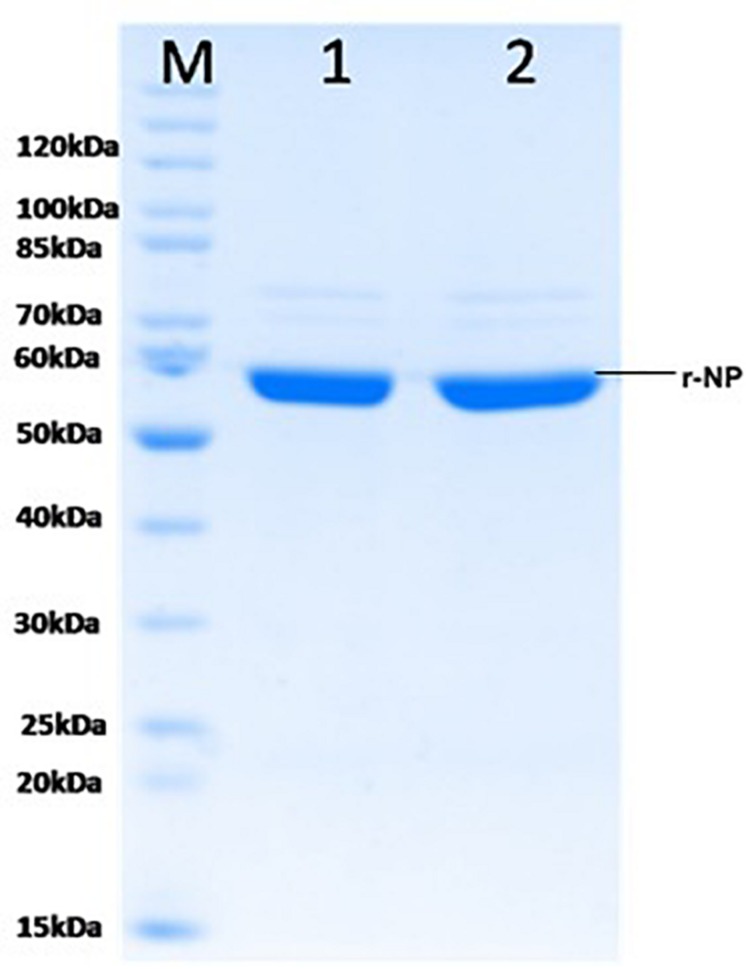
SDS-PAGE analysis of purified r-NP protein. M: Protein molecular weight marker (Cat. # SM0661, Fermentas); lanes 1 and 2, purified recombinant nucleoprotein expressed in BL21 (DE3) cells (56 kDa) using Ni-NTA affinity chromatography.

To maintain the conformational epitopes of recombinant NP, the purification process was carried out employing native conditions. The protein was mixed with Ni-NTA slurry and finally eluted with 250 mM imidazole. The gel analysis revealed a 56-kDa recombinant NP, as a single intense band (Figure 3). The quantity of purified r-NP was 8 mg/L of shake flask culture. The protein r-NP was stored at -80°C to enhance the stability of r-NP in terms of its aggregation and thus functionality.

### Characterization of Recombinant CCHF Nucleoprotein

**FIGURE 4:**
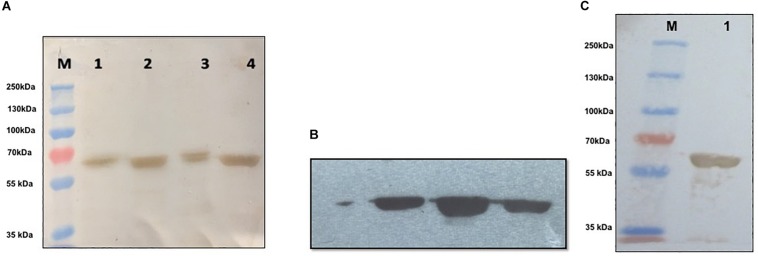
Western blot and chemiluminescent analysis of purified recombinant nucleoprotein (rNP). **(A)** Characterization of purified nucleoprotein of CCHFV. M: Prestained marker (ColorBurst^TM^ Electrophoresis Marker, Cat # C1992, Sigma-Aldrich), lane 1: induced bacterial lysate generated in denaturing condition, lane 2: Purified rNP in insoluble condition, lane 3: induced bacterial lysate generated in soluble condition, lane 4: purified rNP in soluble condition showing immunological reactivity with commercial polyclonal antibody raised in rabbit against CCHFV (Abcam, United States) at 56 kDa. **(B)** Chemiluminescent analysis showing reaction of rNP from three different lots with commercial antibody against CCHFV (Abcam, United States). **(C)** M: Prestained marker (PageRuler^TM^ Plus Prestained Protein Ladder, Cat # 26619, Thermo Fisher Scientific); lane 1: specific binding between rNP and mouse monoclonal anti-His antibody (Sigma-Aldrich, United States).

Recombinant purified NP was characterized using Western blot and MALDI-TOF/MS analysis. In Western blot and chemiluminescent analysis, recombinant NP from both soluble and insoluble fractions was probed with commercial rabbit anti CCHF NP polyclonal antibody and mouse anti-His antibody that showed reactivity at 56-kDa protein band, representing the immunological activity of recombinant NP protein (Figures 4A–C). The recombinant NP was identified by MS/MS analysis of tryptic peptides using MALDI-TOF-TOF. The peak list was searched against the complete non-redundant protein sequence database of NCBI with 16,338,050 sequence entries. The protein was identified as a NP from CCHF virus [gi| 15788941] with a MASCOT score of 637 and a sequence coverage of 20% on MS/MS data (Table 1).

**TABLE 1:** Identification of recombinant nucleoprotein of CCHFV by MS/MS analysis of tryptic peptides using MALDI-TOF-TOF.

**Peptides**	**Observed *m*/*Z* (Da)**	**Mr Experimental**	**Position**	**Ion score**	**RMS Error (ppm)**
LYELFADDSFQQNR	1,745.8160	1,744.8056	359–372	117	2
KLYELFADDSFQQNR	1,873.9119	1,872.9006	358–372	107	2
AQGAQIDTAFSSYYWLYK	2,112.0076	2,111.0000	299–316	119	2
AGVTPETFPTVSQFLFELGK	2,168.1318	2,167.1201	317–336	98	2
GNGLVDTFTNSYSFCESVPNLDR	2,592.1702	2,591.1599	23–45	133	2
TGFNIQDMDIVASEHLLHQSLVGK	2,668.3560	2,667.3327	439–462	70	2

*The protein was identified as nucleoprotein from CCHF virus [gi|15788941] with a MASCOT score of 637 and a sequence coverage of 20% on MS/MS data.*

### Standardization of Indirect IgG ELISA

Crimean-Congo hemorrhagic fever iELISA was standardized with well-characterized antigen, conjugate, and a panel of positive and negative animal sera obtained from Gujarat and Madhya Pradesh. No cross-reactivity was observed with FMD and PPR positive and apparently healthy animal sera. The cutoff of IgG iELISA was set as thrice the average OD value of negative control sera.

### Comparative Evaluation and Statistical Analysis

A panel of 76 suspected samples from different animals (sheep, goat, and bovine) were tested in parallel using in-house iELISA assay and commercial VectoCrimea IgG ELISA kit. The comparative evaluation of both the assays is shown in Table 2.

**TABLE 2:** Comparative evaluation of the in-house CCHF r-NP IgG ELISA with commercially available kit (Vector-Best, Novosibirsk, Russia) revealing true positive, false positive, true negative, and false negative.

**In-house CCHF iELISA**	**Vector-Best Indirect ELISA**		
	**Positive**	**Negative**	**Total**
Positive	27	0	27
Negative	07	42	49

Total	34	42	76

*True positive = 27, true negative = 42, false positive = 0, false negative = 7.*

**FIGURE 5:**
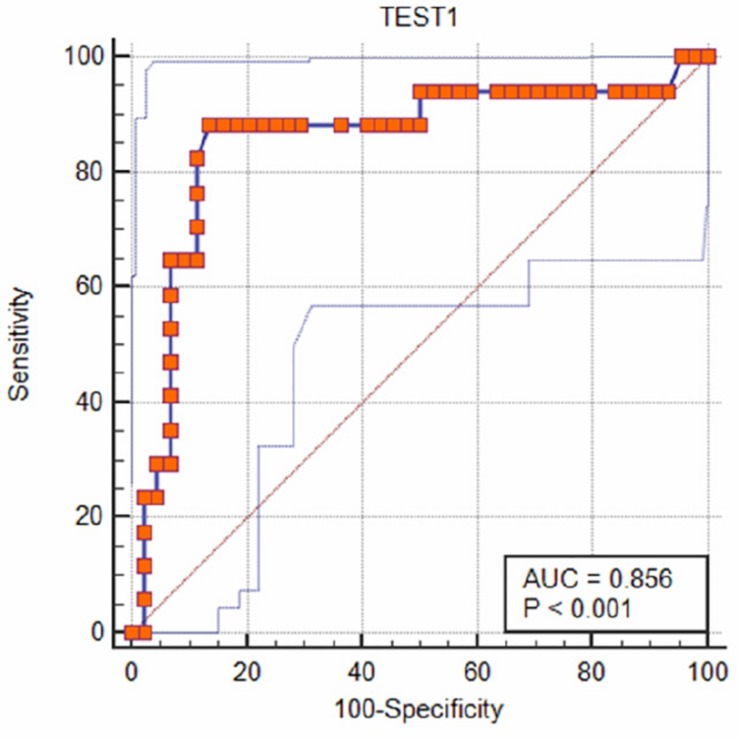
Receiver operating characteristic (ROC) analysis. ROC curve of in-house iELISA was generated with 52 negative sera and 24 positive sera. The area under ROC (AUC) was 0.856 (95% confidence interval). Youden’s index (*J*) for the optimal cutoff was 0.933. Orange dotted blue line represents ROC at 95% confidence interval.

**FIGURE 6:**
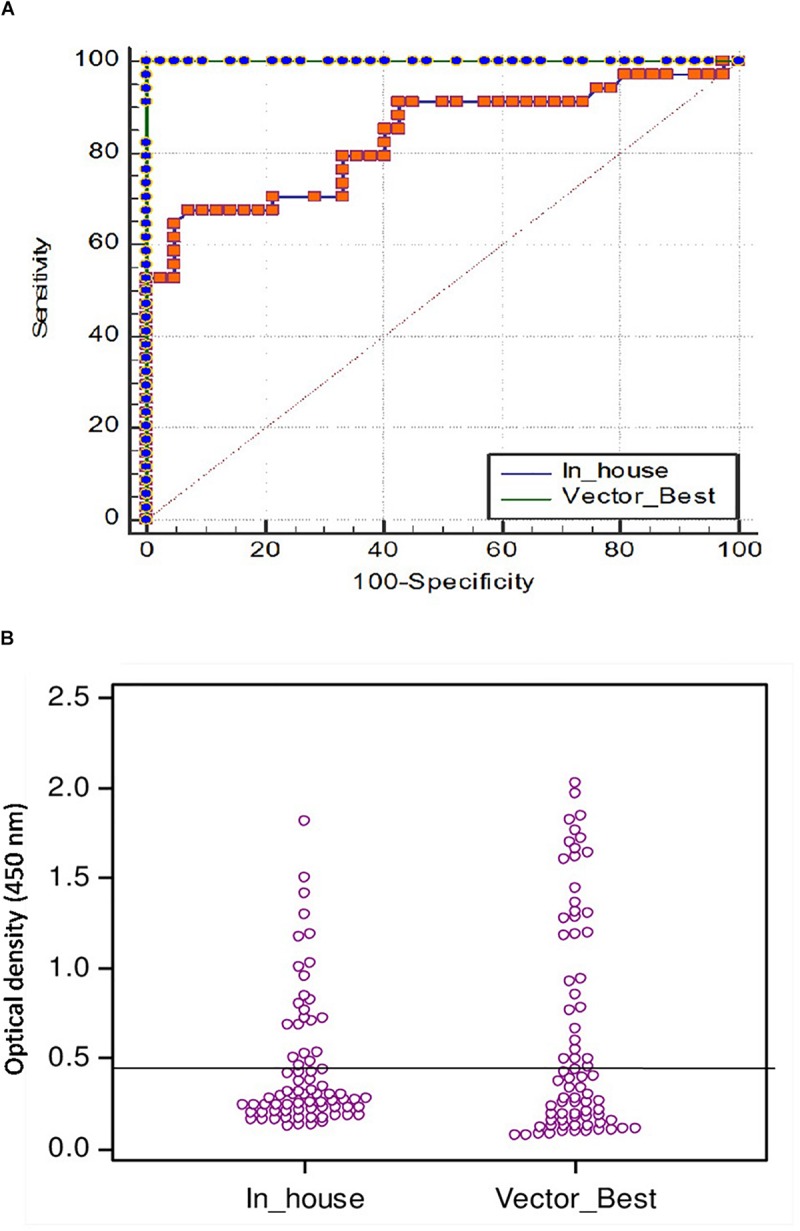
**(A)** Comparison of ROC curves of VectoCrimea IgG ELISA and in-house IgG ELISA using SigmaStat 4.0 with a panel of 76 positive and negative samples. Vector-Best [blue dotted green line; area under the curve (AUC) = 1.00], In-house (Orange dotted blue line; AUC = 0.856). The red dotted line represents the reference diagonal line of non-discrimination and is plotted from point 0,0 to point 1,1. The difference between two curves was 0.166 with a significance value of *p* = 0.0008. **(B)** Threshold of in-house iELISA was determined in comparison to VectoCrimea ELISA assay, estimated as 0.414 at 79.4% sensitivity and 100% specificity.

**FIGURE 7:**
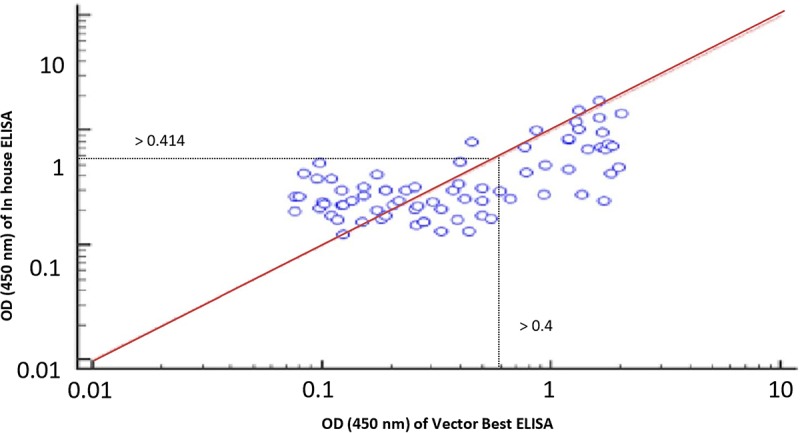
Rho factor was calculated with a Spearman correlation coefficient at 0.78 depicting strong correlation between in-house and commercial assays with 0.414 and 0.4 cutoff of respective assays.

The cutoff was further determined using the ROC curve analysis of in-house iELISA assay and commercial VectoCrimea IgG ELISA assay. The different cutoff was compared in respect to sensitivity and specificity of iELISA. The OD value of 0.414 was recommended as cutoff based on the 95% confidence interval (92.22 to 100%) of in-house assay. The cutoff OD value of 0.4 for the commercial VectoCrimea kit was calculated as per manufacturer instructions. At this threshold value, sensitivity, specificity, PPV, and NPV were calculated. The area under curve (AUC) within ROC showed a value of 0.856 with a Youden’s index (*J*) of 0.933, reflecting a virtuous accuracy of in-house iELISA (Figure 5). The difference between the ROC curves of in-house and Vector-Best was 0.166, with lowest significance level (*p* = 0.0008) (Figure 6A). The comparative evaluation of both the assays indicated a concordance of 90.7%. The sensitivity and specificity of the assay were found to be 79.4 and 100%, while the PPV and NPV of the assay were 100 and 85.7%, respectively (Table 3 and Figure 6B). Rho factor generated through Spearman correlation coefficient using SigmaStat 4.0 was 0.78, whereas the Cohen’s kappa coefficient was found to be 0.748, depicting the strong correlation between the in-house and commercial assays (Figure 7).

**TABLE 3:** Statistical analysis of comparative evaluation of the CCHF r-NP IgG ELISA with commercially available kit (Vector-Best, Novosibirsk, Russia).

**Accordance (%)**	**Sensitivity (%)**	**Specificity (%)**	**PPV (%)**	**NPV (%)**
90.7%	79.4%	100%	100%	85.7%

*True Positive (a) = 27; True Negative (d) = 42; False positive (c) = 00; False negative (b) = 07; Accordance = a + d/a + b + c + d; Sensitivity = a/a + b; Specificity = d/c + d; Positive predictive value = a/a + c; Negative predictive value = d/b + d.*

### Assay Reproducibility

**FIGURE 8:**
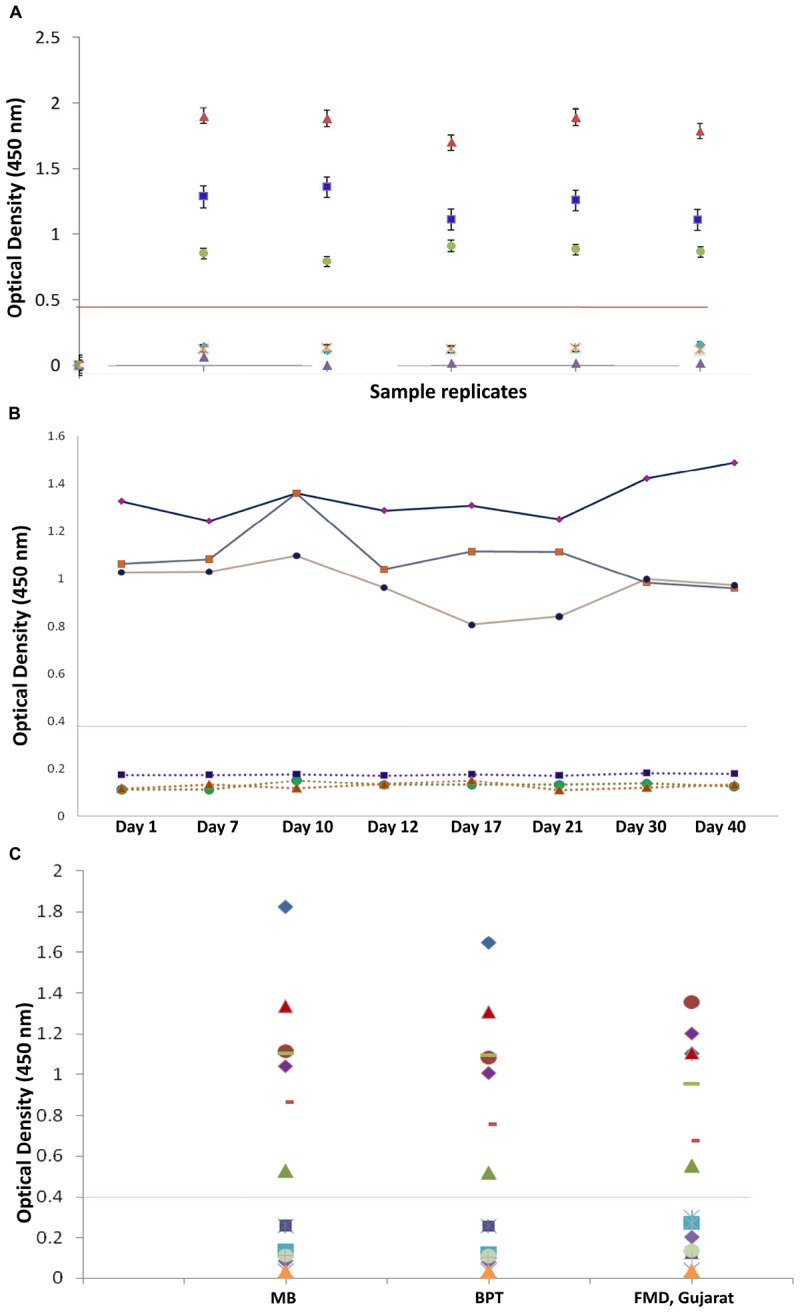
Reproducibility of in-house indirect IgG ELISA. **(A)** Intra-assay variation (variation of replicates in one experiment) of IgG iELISA was performed with a panel of one from each low, medium, and high positive sample and three negative samples and five replicates within a plate. **(B)** Inter-assay-variation (variation (between experiments) performed on eight different days (days 1, 7, 10, 12, 17, 21, 30, and 40) in duplicates by the same operator. **(C)** Inter-operator variation (variation between experiments performed by different operators) and assay precision were conducted at different institutional laboratories and at FMD Centre, Gujarat, with a panel of seven positive and nine negative samples with different operators. Data interpretation was performed with Kruskal–Wallis and one-way ANOVA.)

A panel of calibrators with low, medium, and high IgG positive samples along with negative sera samples were used to study the intra-assay, inter-assay and inter-operator variation analysis. Intra-assay CV was estimated to be 6.4%, depicting the lower level of dispersion around the mean (Figure 8A). Kruskal–Wallis value for intra-assay variation was found to be 0.993, and RM one-way ANOVA *p* value for intra-assay was found to be 0.342, stating no significant difference within the replicates. Kruskal–Wallis value and one-way ANOVA significance was found to be 0.997 and 1.0 for inter-assay variation, respectively, which shows that there is no statistical difference when test was performed in different days (Figure 8B). Percent coefficient of variation for inter-assay was found to be 8.2%.

In the inter-operator assay, the Kruskal–Wallis significance value was 0.835 and one-way ANOVA significance value was 0.983 (Figure 8C). Percent coefficient of variation for inter-operator assay was found to be 14%, which should lie within 15% for inter assay variations. The overall performance of assay is shown in Table 4.

**TABLE 4:** Overall performance of developed in-house IgG iELISA.

**Parameters**	**In-house IgG ELISA**
True positive	27
True negative	42
False positive	00
False negative	07
Sensitivity	79.4%
Specificity	100%
Accordance	90%
AUC	0.856
Youden’s index *J*	0.933
Kruskal–Wallis significance value of intra-assay	0.992
One-way ANOVA significance value of intra-assay	0.342
Percent coefficient of variation of intra-assay	6.2%
Kruskal–Wallis significance value of inter-assay	0.997
One-way ANOVA significance value of inter-assay	1.000
Percent coefficient of variation of inter-assay	8.2%
Kruskal–Wallis significance value of inter-operator assay	0.835
One-way ANOVA significance value of inter-operator assay	0.983
Percent coefficient of variation of inter-operator assay	14%

### Serosurveillance for CCHF in Animal Species

The CCHF iELISA test was applied to screen a total of 640 samples from different domestic ruminants. The result revealed a seropositivity of 17.67% (*n* = 35), 24% (*n* = 60), and 12% (*n* = 23) in sheep, goat, and bovine, respectively. The region (state) and species-wise seropositivity is provided in Table 5.

**TABLE 5:** State and species-wise serosurveillance for CCHF in India.

**State**	**Species of ruminants**	**No. of sample tested**	**No. of sample positive**	**Percent positivity**
Gujarat (*n* = 468)	Sheep	158	30	18.98
	Goat	189	51	26.98
	Bovine	121	20	16.52
Jammu and Kashmir (*n* = 72)	Bovine	72	3	4.16
Rajasthan (*n* = 100)	Sheep	40	5	12.50
	Goat	60	9	15.00

Total		640	118	18.43

## Discussion

Crimean-Congo hemorrhagic fever virus is considered as the one of the most emerging tick-borne viral diseases with recurrent sporadic human cases in various countries of the world. CCHF virus circulates in multiple vertebrate hosts in nature but humans are the dead end host. Hard ticks are the principal vector and infected ticks serve as the lifelong reservoir of the virus. The virus is maintained through transovarial and transstadial transmission. Multiple wild and domestic animals including sheep, goat, and bovine are exposed to the virus through the bite of an infected tick. These ruminants remain asymptomatic, but develop a short viremia followed by seroconversion. CCHFV is maintained through a silent tick–vertebrate–tick enzootic cycle (Spengler et al., 2016). Humans in close contact of these animals or their products are at high risk of acquiring infection. Delayed confirmation of CCHF infection is associated with a risk of nosocomial infection leading to outbreak situations (Aradaib et al., 2010).

In spite of widespread circulation, there is a lack of safe and effective surveillance system in various developing countries (Al-Abri et al., 2017). ELISA is a preferred method for serosurveillance of various diseases in animals. In order to minimize the disease burden in the form of mortality and morbidity, rapid and high-throughput screening is paramount. Availability of a field-based detection system is critical for early and efficient management of CCHF, which is often reported from rural settings. Currently available ELISA systems utilize inactivated virus or suckling mouse brain antigen that poses high biohazard risk. With the advent of recombinant DNA technology, various ELISA systems based on recombinant proteins are developed that provides a safe alternative to infectious viral antigen. In the present study, NP was targeted as a diagnostic intermediate, as it is one of the most important and conserved structural protein of CCHFV. The NP of CCHFV reveals more than 95% conservancy across different genotypes (Hewson et al., 2004; Hawman and Feldmann, 2018). This high conservancy enables applicability of NP from a specific genotype for other genotypes in serodiagnosis testing (Saijo et al., 2002; Emmerich et al., 2018). The NP is also the predominantly expressed antigen after infection and is thus a good candidate for detection of viral antibodies (Schmaljohn and Nichol, 2007).

Though NP has been earlier expressed in several expression systems including baculovirus and prokaryote, its expensive production and instability limit its application for serodiagnosis (Tang et al., 2003; Saijo et al., 2005; Dowall et al., 2012). Therefore, in this study, we have developed a safe, stable, and soluble recombinant NP-based indirect IgG ELISA for serosurveillance. As r-NP does not need any posttranslational modification, a bacterial system was preferred. The recombinant NP was expressed with 6 × histidine tag in *E. coli* BL21 and was purified under native conditions using Ni-NTA affinity chromatography. Since rNP was found in insoluble form when induced at 37°C, the expression was shifted to soluble form by lowering the post-induction temperature to 18°C. This led to the expression of rNP in soluble form, which maintained the conformational integrity of the epitopes. The maintenance of native condition of rNP is crucial as that will enable recognition of antibodies against both linear and conformational epitopes. The high degree of purity (∼95%) and yield (8 mg/L) achieved through shake flask culture in this study shows the potential of the process for commercial exploitation. Further, the immunoreactivity of purified rNP was confirmed using both anti-CCHFV antibody and anti-His antibody in Western blot. The authenticity of expressed rNP was also confirmed through MALDI/TOF-MS. In-house ELISA was optimized through checkerboard titration of antigen, serum, and conjugate with a panel of known positive and negative sera. The cutoff was decided through ROC analysis. Currently, there is no validated commercial serodiagnostic kit available for use in animals. The commercial, Vector-Best kit, which is intended and widely used for human, was adapted in the current study for comparative evaluation of in-house assay as reported earlier (Schuster et al., 2016; Papa et al., 2018; Sas et al., 2018). Further, comparative evaluation of in-house assay and commercial kit was performed, which revealed 90% correlation. The result indicated a diagnostic sensitivity of 79.4% and a diagnostic specificity of 100%, thus minimizing the false-positive result that is crucial in serosurveillance. The stability and performance of in-house assay were also studied with intra-assay, inter-assay, and inter-operator assay variations. In all the validation tests, assay was found to be stable and robust in different sets of conditions such as different days, laboratories, and operators with no statistically significant difference, indicating the robustness and potential utility of in-house iELISA for large-scale applications.

Serosurveillance remains one of the important factors in tracing the circulation of virus in new areas and assists in prevention of infections from animals to human (Bente et al., 2013). This becomes important particularly when there is noprophylaxis and treatment is only supportive for CCHFV. The serosurveillance was carried out using 640 sera from three different ruminant species belonging to three different geographical regions of India. It revealed a seropositivity of 18%, thereby indicating endemicity of CCHFV in India. The relatively higher seropositivity in Gujarat samples is due to the fact that this region is a hotspot for CCHF in India as regular outbreaks have been reported since 2011 (Mourya et al., 2012). The Gujarat samples used in this study were collected from areas reporting human outbreaks with Asia-II lineage (Yadav et al., 2014). The lowest seropositivity in Jammu and Kashmir may be linked to the isolated high-altitude Himalayan region. Interestingly, the successful detection of antibody in Rajasthan samples, where a different lineage (Asia-I) is circulating, clearly demonstrates the utility of in-house iELISA for different lineages (Singh et al., 2016; Yadav et al., 2016). The successful demonstration of the single assay for detection of anti CCHFV antibodies in different animals makes this format amenable for commercial multispecies application. However, this format needs to be further evaluated with a larger sample size from different animals and human.

## Conclusion

The present study led to the development of a process for expression and purification of immunogenic recombinant NP of CCHFV in native conformation. Further, the soluble rNP was explored for the development of a safe, stable, and scalable indirect ELISA that can be used in primary health care facilities to trace the circulation of virus in both human and animals. This assay can also be explored and converted into an on-site point of care testing (POCT) device that eases the diagnosis and surveillance program in resource-limited settings. Further directed and regular serosurveillance programs need to be undertaken in combating the emerging threats of CCHF virus in developing world.

## Data Availability

All datasets generated for this study are included in the manuscript/Supplementary Files.

## Author Contributions

PD conceived the idea. NS performed all the experiments. AS and SN contributed to statistical analysis. JK and SS contributed to sample analysis. SA performed and analyzed MALDI-TOF-TOF. SKS and AK collected and provided suspected animal samples. NS and PD wrote the manuscript. AS and SS critically revised the manuscript. All authors read and approved the final manuscript.

## Conflict of Interest Statement

The authors declare that the research was conducted in the absence of any commercial or financial relationships that could be construed as a potential conflict of interest.

## Supplementary Material

FIGURE S1 **(A)** Induction of *E. coli* BL21 cells at different IPTG concentrations. M: protein molecular weight marker; lane 1: uninduced BL21 culture, lane 2: induction was given at 0.5 mM IPTG, lane 3: induced at 1.0 mM IPTG, lane 4: induced at 1.5 mM IPTG, lane 5: induced at 2.0 mM IPTG, lane 6: induced at 2.5 mM IPTG. **(B)** Induction of BL21 cells at different temperature and time. M: protein molecular weight marker; lane 1: induced BL21 cells for 1 h at 37°C (protein in inclusion bodies), lane 2: induced BL21 cells for 2 h at 37°C (protein in inclusion bodies), lane 3: induced BL21 cells for 3 h at 37°C (protein in inclusion bodies), lane 4: induced BL21 cells for 4 h at 37°C (protein in inclusion bodies), lane 5: induced BL21 cells for 18 h at 18°C (protein in soluble form).

## Abbreviations

ACN: acetonitrile
AUC: area under curve
BSL 4: biosafety level 4
CCHF: Crimean-Congo hemorrhagic fever
CHCA: α-cyano -4-hydroxycinnamic acid
CID: collision-induced dissociation
DAB: 3,3’-diaminobenzidine
DTT: dithiothreitol
FMD: foot-and-mouth disease
H_2_O_2_: Hydrogen peroxide
HRP: horseradish peroxidase
iELISA: indirect enzyme-linked immunosorbent assay
IFA: immunofluorescence
IPTG: isopropyl- β -D-thiogalactopyranoside
MALDI/MS: matrix-assisted laser desorption ionization/mass spectrometry
NP: nucleoprotein
NPV: negative predictive value
PBS: phosphate buffer saline
PMSF: phenyl methyl sulfonyl fluoride
PPR: peste des petits ruminants
PPV: positive predictive value
RE: restriction enzyme
ROC: receiver operating characteristic
SMP: skim milk powder
TFA: trifluoroacetic acid
TIS: timed ion selector.

## References

[B1] Adams M. J. Lefkowitz E. J. King A. M. Q. Harrach B. Harrison R. L. Knowles N. J. (2017). Changes to taxonomy and the international code of virus classification and nomenclature ratified by the international committee on taxonomy of viruses. *Arch. Virol.* 162 2505–2538. 10.1007/s00705-017-3358-5 28434098

[B2] Al-Abri S. S. Abaidani I. A. Fazlalipour M. Mostafavi E. Leblebicioglu H. Pshenichnaya N. (2017). Current status of crimean-congo haemorrhagic fever in the world health organization eastern mediterranean region: issues, challenges, and future directions. *Int. J. Infect. Dis.* 58 82–89. 10.1016/j.ijid.2017.02.018 28259724 PMC7110796

[B3] Álvarez-Rodríguez L. M. Ramos-Ligonio A. Rosales-Encina J. L. Martínez-Cázares M. T. Parissi-Crivelli A. López-Monteon A. (2012). Expression, purification, and evaluation of diagnostic potential and immunogenicity of a recombinant NS3 protein from all serotypes of dengue virus. *J. Trop. Med.* 2012:956875. 10.1155/2012/956875 23258983 PMC3518973

[B4] Aradaib I. E. Erickson B. R. Mustafa M. E. Khristova M. L. Saeed N. S. Elageb R. M. (2010). Nosocomial outbreak of crimean-congo hemorrhagic fever. *Sudan. Emerg. Infect. Dis.* 16:837. 10.3201/eid1605.091815 20409377 PMC2954009

[B5] Babaeipour V. Shojaosadati S. A. Khalilzadeh R. Maghsoudi N. Farnoud A. M. (2010). Enhancement of human γ-interferon production in recombinant E. coli using batch cultivation. *Appl. Biotechnol. Biochem.* 160 2366–2376. 10.1007/s12010-009-8718-5 19655276

[B6] Bente D. A. Forrester N. L. Watts D. M. McAuley A. J. Whitehouse C. A. Bray M. (2013). Crimean-congo hemorrhagic fever: history, epidemiology, pathogenesis, clinical syndrome and genetic diversity. *Antiviral Res.* 100 159–189. 10.1016/j.antiviral.2013.07.006 23906741

[B7] Burt F. J. Leman P. A. Abbott J. C. Swanepoel R. (1994). Serodiagnosis of crimean-congo haemorrhagic fever. *Epidemiol. Infect.* 113 551–562. 10.1017/s0950268800068576 7995364 PMC2271329

[B8] Carter S. D. Surtees R. Walter C. T. Ariza A. Bergeron É Nichol S. T. (2012). Structure, function, and evolution of the crimean-congo hemorrhagic fever virus nucleocapsid protein. *J. Virol.* 86 10914–10923. 22875964 10.1128/JVI.01555-12 PMC3457148

[B9] Casals J. Tignor G. H. (1974). Neutralization and hemagglutination-inhibition tests with crimean hemorrhagic fever-Congo virus. *Proc. Soc. Exp. Biol. Med.* 145 960–966. 10.3181/00379727-145-37933 4818613

[B10] Chan W. T. Verma C. S. Lane D. P. Gan S. K. (2013). A comparison and optimization of methods and factors affecting the transformation of *Escherichia coli*. *Biosci. Rep.* 33:e00086. 10.1042/BSR20130098 24229075 PMC3860579

[B11] Cuzzubbo A. J. Endy T. P. Nisalak A. Kalayanarooj S. Vaughn D. W. Ogata S. A. (2001). Use of recombinant envelope proteins for serological diagnosis of dengue virus infection in an immunochromatographic assay. *Clin. Vaccine Immunol.* 8 1150–1155. 10.1128/cdli.8.6.1150-1155.2001 11687456 PMC96242

[B12] Dowall S. D. Richards K. S. Graham V. A. Chamberlain J. Hewson R. (2012). Development of an indirect ELISA method for the parallel measurement of IgG and IgM antibodies against crimean-congo haemorrhagic fever (CCHF) virus using recombinant nucleoprotein as antigen. *J. Virol. Methods* 179 335–341. 10.1016/j.jviromet.2011.11.020 22155577

[B13] Emmerich P. Mika A. von Possel R. Rackow A. Liu Y. Schmitz H. (2018). Sensitive and specific detection of crimean-congo hemorrhagic fever virus (CCHFV)—Specific IgM and IgG antibodies in human sera using recombinant CCHFV nucleoprotein as antigen in μ-capture and IgG immune complex (IC) ELISA tests. *PLoS Negl. Trop. Dis.* 12:e0006366. 10.1371/journal.pntd.0006366. 29579040 PMC5892944

[B14] Ergönül Ö (2006). Crimean-congo haemorrhagic fever. *Lancet Infect. Dis.* 6 203–214. 10.1016/s1473-3099(06)70435-2 16554245 PMC7185836

[B15] Ge M. Li R. C. Qu T. Gong W. Yu X. L. (2017). Construction of an HRP-streptavidin bound antigen and its application in an ELISA for porcine circovirus 2 antibodies. *AMB Express* 7:177. 10.1186/s13568-017-0473-3 28921455 PMC5603472

[B16] Hawman D. W. Feldmann H. (2018). Recent advances in understanding crimean–congo hemorrhagic fever virus. *F1000Res*. 7:1715. 10.12688/f1000research.16189.1 30416710 PMC6206615

[B17] Hewson R. Chamberlain J. Mioulet V. Lloyd G. Jamil B. Hasan R. (2004). Crimean-congo haemorrhagic fever virus: sequence analysis of the small RNA segments from a collection of viruses worldwide. *Virus Res.* 102 185–189. 10.1016/j.virusres.2003.12.035 15084400

[B18] Huang C. J. Lin H. Yang X. (2012). Industrial production of recombinant therapeutics in *Escherichia coli* and its recent advancements. *Ind. Microbiol. Biotechnol.* 39 383–399. 10.1007/s10295-011-1082-9 22252444

[B19] Liu D. Li Y. Zhao J. Deng F. Duan X. Kou C. (2014). Fine epitope mapping of the central immunodominant region of nucleoprotein from crimean-congo hemorrhagic fever virus (CCHFV). *PLoS One* 9:e108419. 10.1371/journal.pone.0108419 25365026 PMC4217714

[B20] Mertens M. Vatansever Z. Mrenoshki S. Krstevski K. Stefanovska J. Djadjovski I. (2015). Circulation of crimean-congo hemorrhagic fever virus in the former yugoslav republic of macedonia revealed by screening of cattle sera using a novel enzyme-linked immunosorbent assay. *PLoS Negl. Trop. Dis.* 9:e0003519. 10.1371/journal.pntd.0003519 25742017 PMC4351108

[B21] Mostafavi E. Pourhossein B. Esmaeili S. Bagheri Amiri F. Khakifirouz S. Shah-Hosseini N. (2017). Seroepidemiology and risk factors of crimean-congo hemorrhagic fever among butchers and slaughterhouse workers in southeastern Iran. *Int. J. Infect. Dis* 64 85–89. 10.1016/j.ijid.2017.09.008 28935247

[B22] Mourya D. T. Yadav P. D. Shete A. M. Gurav Y. K. Raut C. G. Jadi R. S. (2012). Detection, isolation and confirmation of crimean-congo hemorrhagic fever virus in human, ticks and animals in ahmadabad, india, 2010-2011. *PLoS Negl. Trop. Dis.* 6:e1653. 10.1371/journal.pntd.0001653 22616022 PMC3352827

[B23] Mourya D. T. Yadav P. D. Shete A. M. Sathe P. S. Sarkale P. C. Pattnaik B. (2015). Cross-sectional serosurvey of crimean-congo hemorrhagic fever virus igg in livestock, india, 2013–2014. *Emerg. Infect. Dis.* 21 1837–1839. 10.3201/eid2110.141961 26402332 PMC4593432

[B24] Papa A. Papadopoulou E. Tsioka K. Kontana A. Pappa S. Melidou A. (2018). Isolation and whole-genome sequencing of a crimean-congo hemorrhagic fever virus strain. *Greece. Ticks Tick Borne Dis.* 9 788–791. 10.1016/j.ttbdis.2018.02.024 29525552

[B25] Saijo M. Qing T. Niikura M. Maeda A. Ikegami T. Prehaud C. (2002). Recombinant nucleoprotein based enzyme linked immunosorbent assay for detection of immunoglobulin G to crimean–congo hemorrhagic fever virus. *J. Clin. Microbiol.* 40 1587–1591. 10.1128/jcm.40.5.1587-1591.2002 11980926 PMC130674

[B26] Saijo M. Tang Q. Shimayi B. Han L. Zhang Y. Asiguma M. (2005). Antigen-capture enzyme-linked immunosorbent assay for the diagnosis of crimean-congo hemorrhagic fever using a novel monoclonal antibody. *J. Med. Virol.* 77 83–88. 10.1002/jmv.20417 16032715

[B27] Samudzi R. R. Leman P. A. Paweska J. T. Swanepoel R. Burt F. J. (2012). Bacterial expression of crimean-congo hemorrhagic fever virus nucleoprotein and its evaluation as a diagnostic reagent in an indirect ELISA. *J. Virol. Methods* 179 70–76. 10.1016/j.jviromet.2011.09.023 22001274

[B28] Sas M. A. Comtet L. Donnet F. Mertens M. Vatansever Z. Tordo N. (2018). A novel double-antigen sandwich ELISA for the species-independent detection of crimean-congo hemorrhagic fever virus-specific antibodies. *Antiviral Res.* 151 24–26. 10.1016/j.antiviral.2018.01.006 29330092

[B29] Saxena D. Parida M. Rao P. V. L. Kumar J. S. (2013). Cloning and expression of an envelope gene of west nile virus and evaluation of the protein for use in an IgM ELISA. *Diagn. Microbiol. Infect. Dis.* 75 396–401. 10.1016/j.diagmicrobio.2012.12.007 23357292

[B30] Schmaljohn C. S. Nichol S. T. (2007). *‘Bunyaviridae”‘, in Fields Virology.* Philadelphia: Lippincott Williams & Wilkins, 1741–1789.

[B31] Schuster I. Mertens M. Köllner B. Korytáø T. Keller M. Hammerschmidt B. (2016). A competitive ELISA for species-independent detection of crimean-congo hemorrhagic fever virus specific antibodies. *Antiviral Res.* 134 161–166. 10.1016/j.antiviral.2016.09.004 27623345

[B32] Singh P. Chhabra M. Sharma P. Jaiswal R. Singh G. Mittal V. (2016). Molecular epidemiology of crimean-congo haemorrhagic fever virus in India. *Epidemiol. Infect.* 144 3422–3425. 10.1017/s0950268816001886 27523802 PMC9150195

[B33] Souza I. I. Melo E. S. Ramos C. A. Farias T. A. Osório A. L. Jorge K. S. (2012). Screening of recombinant proteins as antigens in indirect ELISA for diagnosis of bovine tuberculosis. *Springer plus* 1:77. 10.1186/2193-1801-1-77 23419946 PMC3569591

[B34] Spengler J. R. Estrada-Peña A. Garrison A. R. Schmaljohn C. Spiropoulou C. F. Bergeron É (2016). A chronological review of experimental infection studies of the role of wild animals and livestock in the maintenance and transmission of crimean-congo hemorrhagic fever virus. *Antiviral Res.* 135 31–47. 10.1016/j.antiviral.2016.09.013 27713073 PMC5102700

[B35] Tang Q. Saijo M. Zhang Y. Asiguma M. Tianshu D. Han L. (2003). A patient with crimean-congo hemorrhagic fever serologically diagnosed by recombinant nucleoprotein-based antibody detection systems. *Clin. Vaccine Immunol.* 10 489–491. 10.1128/cdli.10.3.489-491.2003 12738657 PMC154974

[B36] Vagenende V. Yap M. G. S. Trout B. L. (2009). Mechanisms of protein stabilization and prevention of protein aggregation by glycerol. *Biochemistry* 48 11084–11096. 10.1021/bi900649t 19817484

[B37] Vanhomwegen J. Alves M. J. Županc T. A. Bino S. Chinikar S. Karlberg H. (2012). Diagnostic assays for crimean-congo hemorrhagic fever. *Emerg. Infect. Dis.* 18 1958–1965. 10.3201/eid1812.120710 23171700 PMC3557897

[B38] Yadav P. Patil D. Y. Shete A. M. Kokate P. Goyal P. Jadhav S. (2016). Nosocomial infection of CCHF among health care workers in rajasthan. india. *BMC Infect. Dis.* 16:624. 10.1186/s12879-016-1971-7 27809807 PMC5094004

[B39] Yadav P. Raut C. Patil D. Majumdar T. D. Mourya D. T. (2014). Crimean-congo hemorrhagic fever: current scenario in india. *Proc. Natl. A. Sci. India Sec B Biol. Sci.* 84 9–18. 10.1007/s40011-013-0197-3 PMC7100343 32226205

[B40] Zivcec M. Scholte F. Spiropoulou C. Spengler J. Bergeron É. (2016). Molecular insights into crimean-congo hemorrhagic fever virus. *Viruses* 8:106. 10.3390/v8040106 27110812 PMC4848600

