# Supplementary figures and images for: Development of Multispecies Recombinant Nucleoprotein-Based Indirect ELISA for High-Throughput Screening of Crimean-Congo Hemorrhagic Fever Virus-Specific Antibodies
Source: Front Microbiol. 2019 Aug 23;10:1822. doi: 10.3389/fmicb.2019.01822 (PMC6716110; doi:10.3389/fmicb.2019.01822)

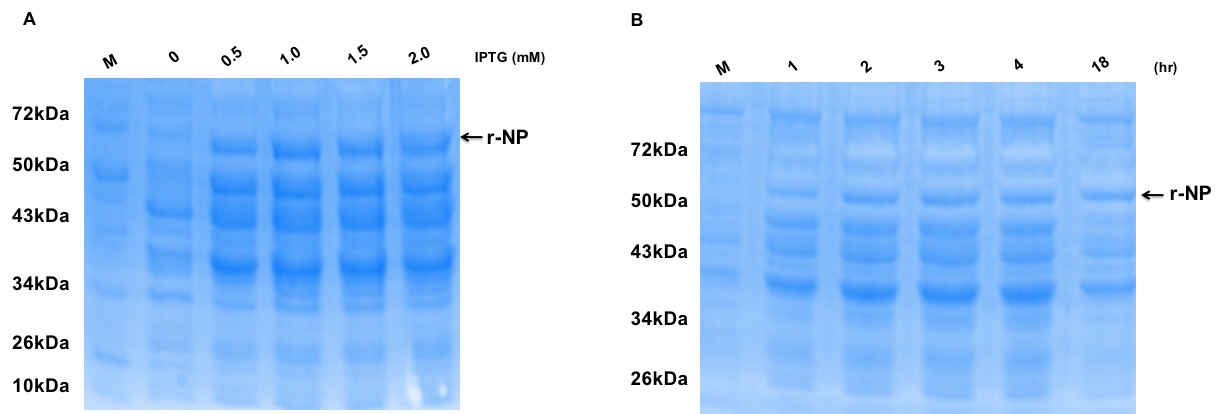

Supplement: FIGURE S1 — (A) Induction of E. coli BL21 cells at different IPTG concentrations. M: protein molecular weight marker; lane 1: uninduced BL21 culture, lane 2: induction was given at 0.5 mM IPTG, lane 3: induced at 1.0 mM IPTG, lane 4: induced at 1.5 mM IPTG, lane 5: induced at 2.0 mM IPTG, lane 6: induced at 2.5 mM IPTG. (B) Induction of BL21 cells at different temperature and time. M: protein molecular weight marker; lane 1: induced BL21 cells for 1 h at 37°C (protein in inclusion bodies), lane 2: induced BL21 cells for 2 h at 37°C (protein in inclusion bodies), lane 3: induced BL21 cells for 3 h at 37°C (protein in inclusion bodies), lane 4: induced BL21 cells for 4 h at 37°C (protein in inclusion bodies), lane 5: induced BL21 cells for 18 h at 18°C (protein in soluble form). [file Image_1.JPEG]
